# Autoinducers Act as Biological Timers in *Vibrio harveyi*

**DOI:** 10.1371/journal.pone.0048310

**Published:** 2012-10-26

**Authors:** Claudia Anetzberger, Matthias Reiger, Agnes Fekete, Ursula Schell, Nina Stambrau, Laure Plener, Joachim Kopka, Phillippe Schmitt-Kopplin, Hubert Hilbi, Kirsten Jung

**Affiliations:** 1 Munich Center for integrated Protein Science at the Department of Biology I, Microbiology, Ludwig-Maximilians-Universität Munich, Germany; 2 Technische Universität Munich, Chair of Analytical Food Chemistry, Freising, Germany; 3 Max von Pettenkofer-Institut, Ludwig-Maximilians-Universität Munich, Munich, Germany; 4 Max-Planck-Institute for Molecular Plant Physiology, Potsdam-Golm, Germany; Arizona State University, United States of America

## Abstract

Quorum sensing regulates cell density-dependent phenotypes and involves the synthesis, excretion and detection of so-called autoinducers. *Vibrio harveyi* strain ATCC BAA-1116 (recently reclassified as *Vibrio campbellii*), one of the best-characterized model organisms for the study of quorum sensing, produces and responds to three autoinducers. HAI-1, AI-2 and CAI-1 are recognized by different receptors, but all information is channeled into the same signaling cascade, which controls a specific set of genes. Here we examine temporal variations of availability and concentration of the three autoinducers in *V. harveyi*, and monitor the phenotypes they regulate, from the early exponential to the stationary growth phase in liquid culture. Specifically, the exponential growth phase is characterized by an increase in AI-2 and the induction of bioluminescence, while HAI-1 and CAI-1 are undetectable prior to the late exponential growth phase. CAI-1 activity reaches its maximum upon entry into stationary phase, while molar concentrations of AI-2 and HAI-1 become approximately equal. Similarly, autoinducer-dependent exoproteolytic activity increases at the transition into stationary phase. These findings are reflected in temporal alterations in expression of the *luxR* gene that encodes the master regulator LuxR, and of four autoinducer-regulated genes during growth. Moreover, *in vitro* phosphorylation assays reveal a tight correlation between the HAI-1/AI-2 ratio as input and levels of receptor-mediated phosphorylation of LuxU as output. Our study supports a model in which the combinations of autoinducers available, rather than cell density *per se*, determine the timing of various processes in *V. harveyi* populations.

## Introduction

The term “quorum sensing”, introduced by Peter Greenberg in 1994 [1], refers to a concept according to which bacteria constantly produce and excrete low-molecular-weight signaling molecules, called autoinducers (AIs), into the medium. As cell numbers increase, so does the concentration of AIs. At a defined threshold AI concentration, the population expresses a synchronized, AI-specific response – usually a phenotype, such as virulence, light production or biofilm formation, which is more effective when deployed by a group of cells rather than a single bacterium.

**Figure 1:**
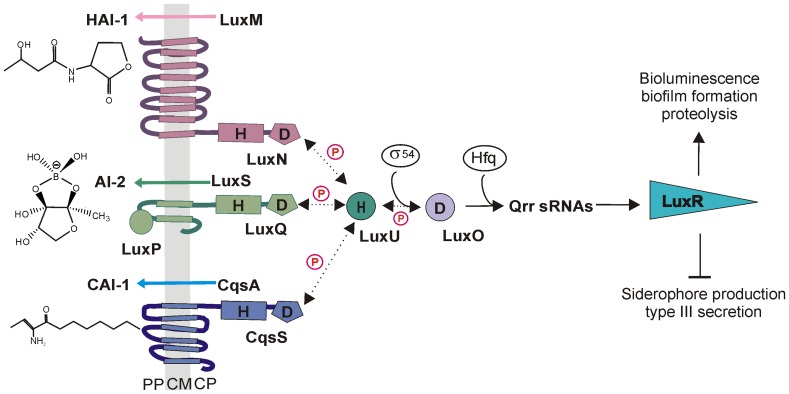
The quorum sensing circuit in *Vibrio harveyi*. In *V. harveyi* the three autoinducers HAI-1, AI-2 and CAI-1 are synthesized by the synthases LuxM, LuxS and CqsA. The cognate hybrid sensor kinases LuxN, LuxQ together with LuxP, and CqsS detect each autoinducer and effectively measure their concentrations: the higher the autoinducer concentration, the lower is the autophosphorylation activity of the hybrid kinases. The phosphoryl groups are transferred via phosphorelay including the histidine phosphotransfer protein LuxU to the σ^54^-dependent transcriptional activator LuxO. Phosphorylated LuxO in turn activates transcription of five regulatory sRNAs, four of which (Qrr1-4) are active. Together with the RNA chaperone Hfq, these sRNAs destabilize the transcript that codes for the master regulator LuxR. The LuxR content is further regulated by additional feedback regulation (see text for details). Autoinducers activate genes required for bioluminescence, biofilm formation and proteolysis and repress genes involved in type III secretion and siderophore production. Dashed lines indicate phosphotransfer reactions. *H* (histidine) and *D* (aspartate) denote the phosphorylation sites. *CM*, cytoplasmic membrane; *CP*, cytoplasm; *PP*, periplasm.

*Vibrio harveyi* strain ATCC BAA-1116 (recently reclassified as *Vibrio campbelli* [2], [3]), one of the best studied model organisms for quorum sensing, produces and responds to three different classes of AIs: the species-specific HAI-1 [N-(3-hydroxybutyryl)-homoserine lactone], AI-2 (furanosyl borate diester), which is synthesized by many bacterial species, and the genus-specific CAI-1 [*(Z)*-3-aminoundec-2-en-4-one (Ea-C8-CAI-1)] [4]–[7]. These three AIs are recognized by the three membrane-bound hybrid sensor kinases LuxN, LuxQ (in cooperation with the periplasmic AI-2-binding protein LuxP) and CqsS respectively (Fig. 1) [5], [6], [8]–[10]. Information on AI concentrations is transduced by the sensor kinases via phosphorelay to the histidine phosphotransfer protein LuxU and further to the response regulator LuxO [11]. Recently, a new circuit consisting of the soluble histidine kinase HqsK and the NO-sensing H-NOX was reported, which feeds its information into the network at the level of LuxU [12]. At low cell densities (low AI concentration) phosphorylated LuxO activates the transcription of five small regulatory RNAs; four of these (Qrr1-4), together with the RNA chaperone Hfq, act to destabilize the *luxR* transcript [13]. At high cell densities (high AI concentration) LuxO is dephosphorylated and LuxR is produced [11]. A direct inhibitory effect of HAI-1 on the kinase activity of LuxN has already been demonstrated [14]. LuxR in turn activates and represses large numbers of genes [15]. At high AI concentrations, genes involved in bioluminescence [16], biofilm formation [17] and extracellular proteolysis [18] are induced, while genes for type III secretion [19] and siderophore production [20] are repressed.

Several feedback loops are known to regulate the content of LuxR in the cells. These involve autorepression of *luxR* [21], activation of *qrr2-4* transcription by LuxR [22], autorepression of *luxO* and repression of *luxO* translation by Qrr sRNAs [23], repression by AphA, a recently described antagonist of LuxR [24], and down-regulation of *luxMN* translation by Qrr sRNAs [25].

In spite of detailed knowledge of the complex signaling cascade, it is still unclear why *V. harveyi* produces three AIs but channels all information into a single signaling cascade. Moreover, we have previously shown that extracellular concentrations of AIs correlate with the degree of cell-to-cell variance in the expression of bioluminescence [17]. We have therefore examined the pattern of accumulation of the three AIs in a growing culture of the wild type strain, from the early exponential (10^6^ cells*mL^−1^, OD_600_ = 0.001) to the stationary growth phase (2*10^9^ cells*mL^−1^, OD_600_ = 2). It should be noted here that, in previous studies, the expression of AI-regulated genes has been analyzed predominantly by studying their responses to exogenously provided AIs [18], [26]. We have also monitored the time course of *luxR* transcript levels and diverse AI-regulated processes. Our data suggest a model in which the precise composition of the AIs present in certain growth phases, rather than the cell density *per se*, is the more important influence on AI-regulated gene expression. This model is supported by *in vitro* phosphorylation studies.

## Materials and Methods

### Strains and growth conditions

The *V. harveyi* strains listed in Table 1 were cultivated in autoinducer bioassay (AB) medium [27], and incubated aerobically on a rotary shaker at 30°C. When necessary, the medium was supplemented with chloramphenicol (33 µg*mL^−1^). Overnight cultures were diluted 5,000-fold into fresh AB medium and grown for a further 20 h. Samples were taken every hour, and cells were removed by centrifugation at 5,000× g for 15 min. The culture fluids were then filtered (0.20 µm) and stored at −20°C or used immediately. To measure the cell density of a *V. harveyi* culture the optical density at 600 nm was determined for values larger than 0.01. For cultures with an OD_600_<0.01 the number of colony-forming units was determined directly, and the optical density was calculated (OD_600_ = 1 corresponds to 10^9^ cells*mL^−1^).

**Table 1:** Strains and plasmids used in this study.

Strain or plasmid	Genotype or description	Reference
*V. harveyi* BB120	wild type ATCC BAA-1116	[64]
*V. harveyi* MM77	*luxM*::Tn*5*, *luxS::*Cm^r^	[18]
*V. harveyi* JAF78	Δ*luxO::*Cm^r^	[11]
*V. harveyi* JAF548	*luxO*-D47E	[11]
*V. harveyi* JMH634	Δ*luxM*, Δ*luxS*, *cqsA::Cm^r^*	[8]
*V. harveyi* JMH626	Δ*luxN*, *luxQ*::Tn5, *cqsA::Cm^r^*	[8]
*V. cholerae* MM920	Δ*cqsA*, Δ*luxQ*, pBB1	[33]
*E. coli* TKR2000	Δ*kdpFABCDE thi rha lacZ nagA trkA405 trkD1 atp706*	[65]
*E. coli* MDAI-2	*luxS::*Tet^r^-derivative of *E. coli* W3110	[66]
*E. coli* JM109	*recA1 endA1 gyrA96 traD36 thi hsdR17 supE44* λ*^−^ relA1* Δ*(lac-proAB)/*F' *proA* ^+^ *B* ^+^ *lacI* ^q^ *lacZ*ΔM15	[67]
pPV5-1	*kdpD* in pKK223-3	[29]
pPV5-10	pPV5-1 with KpnI site after the start codon of *kdp*	This work
pNKN	*luxN* in pPV5-10	This work
pNKQ	*luxQ* in pPV5-10	This work
pGEX_LuxP	*luxP* in pGEX-4T1	[10]
pQE30LuxU-6His	*luxU* in pQE30	[14]
pQE30LuxS-6His	*luxS* in pQE30	[41]
pQE30Pfs-6His	*pfs* in pQE30	[41]
pTS-6	*cqsA* in pGEM-T-Easy	[34]

*Escherichia coli* strains listed in Table 1 were grown in lysogenic broth (LB) [28] or KML medium [1% (w/v) tryptone, 1% (w/v) KCl, 0.5% (w/v) yeast extract] and incubated aerobically in Erlenmeyer flasks on a rotary shaker at 37°C. When necessary, the medium was supplemented with ampicillin (100 µg*mL^−1^) or chloramphenicol (33 µg*mL^−1^).

### Cloning of luxN and luxQ

For overexpression of *luxN* and *luxQ* in *E. coli* TKR2000 each gene was inserted into plasmid pKK223-3, in which expression is under control of the *tac* promoter. To use the natural Shine Dalgarno box of *kdpD*, plasmid pPV5-1 (*kdpD* in pKK223-3 [29]) was used, and *kdpD* was replaced by *luxN* or *luxQ*. For ease of cloning, a KpnI site was first inserted downstream of the start codon of *kdpD* by two-step PCR [30] resulting in plasmid pPV5-10. *luxN* and *luxQ* were amplified from genomic DNA by PCR using the primer pairs LuxN/KpnIsense and LuxN/HindIIIantisense, and LuxQ/KpnIsense and LuxQ/HindIIIantisense. The PCR fragments were restricted with KpnI and HindIII and cloned into plasmid pPV5-10 to obtain plasmids pNKN and pNKQ. Sequences of the primers used are available on request.

### Preparation of inverted membrane vesicles

*E. coli* TKR2000 was transformed with plasmids pNKN and pNKQ, encoding wild type LuxN and LuxQ. Each protein carried a His-tag at the C-terminus, attached either directly (LuxQ) or via a two-amino acid linker (LeuGln, LuxN). Inside-out membrane vesicles were prepared as described [14].

### Heterologous production of LuxP and LuxU

LuxP was produced in and purified from *E. coli* MDAI-2 transformed with the plasmid pGEX_LuxP as described before [10]. LuxU was produced and purified exactly as described before, using *E. coli* JM109 transformed with plasmid pQE30LuxU-6His [14]. All proteins were stored at −80°C prior to use.

### Analytical procedures

Protein concentrations were determined by the method of Peterson [31] using bovine serum albumin as standard. Proteins were fractionated by SDS-PAGE [32]. His-tagged Lux proteins on immunoblots were labeled with mouse monoclonal antibodies directed against the His-tag (Qiagen) and detected by incubation with alkaline phosphatase-conjugated anti-mouse IgG (GE Healthcare) according to the manufacturer's instructions. Quantitative Western blots were scanned with 300 dpi resolution in 256 gray scales and imported as TIFF files into ImageQuant 5.0 (GE Healthcare). The amount of Lux proteins associated with membrane vesicles was quantified by comparison with the total amount of purified His-tagged LuxN.

### Determination of autoinducer concentrations in cell-free culture medium

HAI-1 was quantified by UPLC using an Acquity UPLC System with a 2996 PDA detector controlled by Empower software (Waters). The system was equipped with an Acquity 2.1×100 mm BEH C18 column packed with 1.7-µm particles (Waters), which was maintained at a constant temperature of 60°C. The Sample Manager was kept at 27°C. Aliquots (5 µl) of sample were injected via a partial loop with needle overfill, and all samples were analyzed three times. Water (Biosolve) containing 3% acetonitrile (Biosolve) served as the mobile phase, and isocratic elution was applied at a flow rate of 0.9 mL*min^−1^ causing a back-pressure of 770 bar. Detection was performed at 195 nm with a scan rate of 20 Hz. The analysis time for each injection was set to 3 min, and all sample constituents were eluted from the column. The retention time for HAI-1 (0.579 min) and the UV-Vis spectra of the peak provided the criteria for identification of the compound and assessment of its purity. A standard solution of HAI-1 was used for calibration and quantification of the analyte.

**Figure 2:**
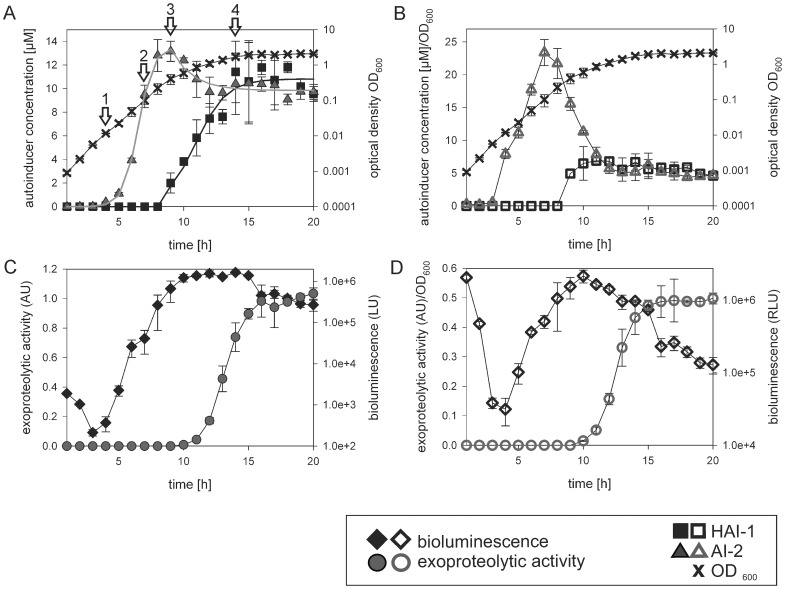
Time course of HAI-1 and AI-2 production (A, C), bioluminescence and exoproteolytic activity (B, D) during growth of *V. harveyi*. Cells of an overnight culture of *V. harveyi* BB120 were diluted 5,000-fold in fresh AB medium and cultivated aerobically at 30°C. Samples were taken at the times indicated and autoinducer concentrations in the medium, bioluminescence levels and exoproteolytic activity were determined. (A, B) Extracellular HAI-1 concentrations were determined by UPLC (black squares). AI-2 was captured with the binding protein LuxP, and quantified by bioassay (gray triangles). In parallel, the CFU and the optical density (OD_600_, black crosses) were determined. Closed symbols (A) indicate the extracellular concentrations of the autoinducers. Open symbols (B) indicate autoinducer concentrations normalized relative to the OD_600_ value. The arrows (A) mark the time points chosen for transcriptional analysis (see Fig. 6). (C, D) The same samples were analyzed for bioluminescence level (light units, LU) and exoproteolytic activity (AU). Closed symbols (C) indicate bioluminescence levels (black diamonds) and exoproteolytic activity (gray circles) as absolute values; open symbols (D) are normalized to the corresponding optical density. All experiments were performed in triplicate and error bars indicate standard deviations of the mean.

To determine the concentration of AI-2 in culture fluids, LuxP-GST (2.5 mg*mL^−1^) was added, and the mixture was incubated at 30°C for 30 min. The LuxP-AI-2 complex was then separated from the culture fluid by centrifugation of the sample through a NMWL filter (Millipore) with a 30,000-dalton cut-off, so that the protein-AI-2 complex was retained on the filter. To dissociate the complex, the membrane was washed in water, and the extract was kept at 50°C for 10 min and filtered again. The filtrate containing the AI-2 molecules was subsequently used in a bioluminescence assay with *V. harveyi* MM77 as reporter strain. To obtain a calibration curve, standard solutions of synthetic AI-2 (0 to 50 μM) were tested in the reporter assay with *V. harveyi* MM77 (see below). The fitted lines for the HAI-1 and AI-2 concentrations presented in Figure 2 were generated using the following equations:

HAI-1: 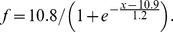



AI-2: 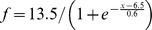 for 1 to 9 h and 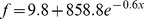 for 9 to 20 h.

**Figure 3:**
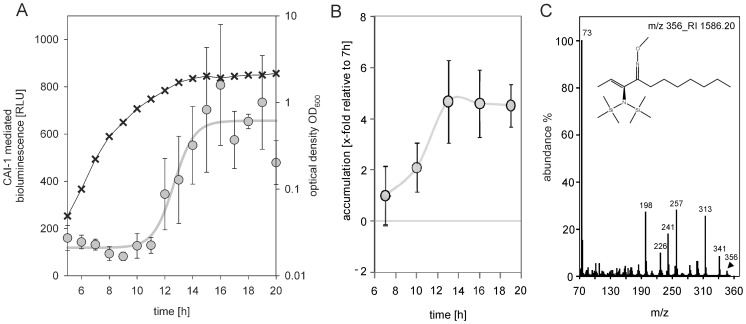
Alterations in CAI-1 activity during growth of *V. harveyi*. (A) CAI-1 activity was determined in cell-free culture fluids (the same samples as described in Fig. 2) using a bioassay with *V. cholerae* MM920 as reporter strain. Levels of CAI-1 mediated bioluminescence are indicated by light gray dots. A curve is presented to guide the eye. The optical density (OD_600_) is plotted as crosses. All experiments were performed at least in triplicate, and error bars indicate standard deviations of the mean. (B, C) Unbiased GC-TOF-MS profiling was used to identify signaling molecules that accumulated in the medium after 7 h of growth. (B) Single-ion responses with defined retention indices (RI) close to that expected for Ea-C8-CAI-1 were tested for significant increases between 7 h and 19 h of cultivation, p<1.0 10^−4^. The data were presented as x-fold accumulation in comparison to the 7 h time point. The replicate mass spectrum and respective retention index may be retrieved from the Golm Metabolome Database (http://gmd.mpimp-golm.mpg.de/) using the identifier code A158016 (m/z 356_RI 1586.20). (C) Representative mass spectrum of candidate signaling molecules possibly representing Ea-C8-CAI-1. The mass of compound A158016 corresponds to Ea-C8-CAI-1 (arrow), which was modified with trimethylsilylated methoxyamine. Its chemical structure is shown. All experiments were performed at least in triplicate. Error bars indicate standard deviations of the mean. Time courses were interpolated by smoothed lines using MS-EXCEL software.

CAI-1 levels in cell-free cultures fluids of wild type *V. harveyi* were determined using the *V. cholerae* reporter strain MM920 [33], [34] or the *V. harveyi* reporter strain JMH626 in a bioluminescence assay, incubating a fresh diluted culture of the reporter strain with cell-free culture fluids [50% (v/v)]. In parallel, CAI-1 was analyzed by GC-TOF-MS. Metabolites in culture fluids prepared as described above were chemically modified by sequential methoxyamination and trimethylsilylation, as described earlier [35], [36]. Gas chromatography coupled to electron impact ionization-time of flight-mass spectrometry was performed using an Agilent 6890N24 gas chromatograph coupled to a Pegasus III time-of-flight mass spectrometer (LECO, St. Joseph, USA). Chromatograms were acquired with CHROMATOF software 1.00, Pegasus driver 1.61 (Leco; http://www.leco.de). Selective ion traces and peak heights were extracted from the NetCDF CHROMATOF export, and processed using the TagFinder software [37]. Compounds that accumulated (relative to their levels in the sample taken after 7 h of cultivation) were filtered according to significance (p) using Students t-test and the Kruskal-Wallis test. The mass spectrum of modified CAI-1 was generated under manual supervision by automated deconvolution (CHROMATOF software 1.00). Replicate mass spectra and retention indices [38] were uploaded to the Golm Metabolome Database, http://gmd.mpimp-golm.mpg.de [39], [40]. Available compound information may be retrieved from http://gmd.mpimp-golm.mpg.de/search.aspx using the “A” identifier code (see legend to Fig. 3). The fitted line for the CAI-1 concentration presented in Figure 3 was generated using the following equation: 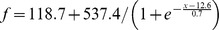.

### Synthetic autoinducers

HAI-1 (D- and L-isomers) was purchased from the University of Nottingham and dissolved in a minimal volume of acetonitrile [10% (v/v)], diluted with water to a concentration of 100 mM and stored at −20°C. DPD, the precursor of AI-2, was synthesized *in vitro* using S-adenosyl-homocysteine (Sigma-Aldrich) and the enzymes LuxS and Pfs, followed by purification over boric acid resin [41]. LuxS and Pfs were produced heterologously in *E. coli* JM109 transformed with plasmid pQE30LuxS-6His or pQE30Pfs-6His, respectively, and purified as described before [41], [42]. Purity and yield of AI-2 were indirectly determined as described [43] using the method of Ellman [44]. The biological activities of both AIs were determined using the bioluminescence based reporter assay [45] and *in vitro* phosphorylation experiments with LuxN/LuxQ and LuxU.

### Bioluminescence assay

Luminescence produced by *V. harveyi* strains was determined in microtiter plates in a Centro LB960 (Berthold Technology) for 0.1 s, and data are reported as light units (LU) or relative light units (RLU) [counts*s^−1^] per OD_600_ unit. All measured data were below the saturation range of the instrument (2.2*10^6^ LU). To determine the dose-dependent effect of HAI-1 or AI-2, strain MM77 (*luxM*::Tn*5 luxS*::Cm^r^) was used as reporter. Overnight cultures of strain MM77 were diluted 1∶100 in AB medium containing culture fluids [50% (v/v)] or various concentrations of synthetic HAI-1 and AI-2. Cells were grown until the mid-exponential growth phase and analyzed as described above.

### Protease assay

Exoproteolytic activity of *V. harveyi* strains was measured by incubating hide powder azure (Sigma-Aldrich) in phosphate-buffered saline (PBS, pH 7.2) with cell-free culture fluids at 37°C. The reaction was stopped with trichloracetic acid [6.7% (v/v)] after 2 h, and the absorbance at 600 nm was measured [46]. The activity is expressed as the difference between initial and final absorption after 2 h (AU). The assay was adapted to microtiter plates using 0.5 mg hide powder azure, 100 μl PBS and 100 μl culture fluid per well. For standardization, protease K (Sigma-Aldrich) was used. When indicated the metalloprotease inhibitor EDTA (5 mM) and the serine protease inhibitor phenylmethylsulfonyl fluoride (PMSF) (1 mM) were added prior to incubation [47].

### Kinetic analysis of the transcriptional response of AI-induced/repressed genes by qRT-PCR

*V. harveyi* strains BB120 and JMH634 were cultivated as described above. Samples were withdrawn, and RNA was isolated as described before [48]. The RNA was then used as template for random-primed first-strand cDNA synthesis according to the manufacturer's instructions. Quantitative real-time PCR (qRT-PCR) (iQ5 real-time PCR detection system, Biorad) was performed using the synthesized cDNA, a SYBR-green detection system (Biorad) and specific internal primers for *luxA, luxR*, *vhpA, vopN, vscP* and *recA*. Duplicate samples from three independent biological experiments were used, and the C_T_ value (cycle threshold) was determined after 40 cycles using the iQ software (Biorad). Values were normalized with reference to *recA* and relative changes in transcript levels were calculated using the comparative C_T_ method [49].

### Phosphorylation and dephosphorylation assays

Phosphorylation reactions were performed in phosphorylation buffer (50 mM Tris/HCl pH 8.0, 10% (v/v) glycerol, 500 mM KCl, 2 mM DTT) at 25°C. The sensor kinases LuxQ and LuxN were tested as full-length membrane integrated proteins in inverted membrane vesicles. To incorporate LuxP into LuxQ-bearing membrane vesicles, vesicles were subjected to three cycles of freezing and thawing.

A typical reaction mixture for a phosphorylation assay (total volume 150 μl) contained 7.5 mg*mL^−1^ (LuxQ) or 5 mg*mL^−1^ (LuxN) membrane proteins, and 0.36 mg*mL^−1^ purified LuxU. LuxP was added at a concentration of 0.96 mg*mL^−1^. For experiments involving both kinases, the concentration of each kinase was halved. The reaction was started by addition of radiolabeled Mg^2+^-ATP, typically 100 μM [γ-^32^P] ATP (specific radioactivity of 0.94 Ci*mmol^−1^; Perkin Elmer) and 110 μM MgCl_2_. At various times thereafter, the reaction was terminated by the addition of SDS loading buffer [32], followed by separation of the proteins on SDS-polyacrylamide gels. Gels were dried at 80°C on filter paper, exposed to a phosphoscreen for at least 24 h, and subsequently scanned using a PhosphorImagerSI (GE Healthcare). Different dilutions of [γ-^32^P] ATP were used to generate a calibration curve for quantification of the signal intensities of phosphorylated proteins using ImageQuant software (Molecular Dynamics V5.0; GE Healthcare). All enzymatic activities were calculated as mean values of at least three independent experiments. The gels shown are representative of each set of experiments.

For dephosphorylation assays LuxU was first phosphorylated using LuxN. In this case, the reaction mixture contained twice the usual amounts of LuxN and LuxU. After 10 min of incubation, membrane vesicles were removed by centrifugation (100,000 x *g*, 15 min, 4°C), and ATP was removed by gel filtration (Sephadex G25 columns, GE Healthcare). Dephosphorylation of phosphorylated LuxU (0.18 mg*mL^−1^) was then initiated by the addition of 110 μM MgCl_2_ and membrane vesicles containing LuxQ (3.75 mg*mL^−1^). Phosphorylated LuxU was quantified as described above.

## Results

### Patterns of accumulation of the three autoinducers change during growth of V. harveyi

The extracellular concentrations of the three AIs were determined in a wild type population of *V. harveyi* (strain BB120, now *V. campbellii* ATCC BAA-1116) grown in liquid AB medium at regular intervals (Fig. 2). To start the experiment, a dense inoculum from an overnight culture was diluted 1∶5,000 into fresh medium at time 0. The concentration of the furanosylborate diester AI-2 increased rapidly, whereas the acyl-homoserine lactone HAI-1 remained undetectable for the first 8 h (Fig. 2A; the detection limit for HAI-1 using UPLC was 0.5 μM). The concentration of AI-2 reached a maximum of 13.2±0.8 μM near the end of the exponential growth phase, decreased thereafter to about 10 μM (9.6±0.6 μM) and remained constant at this concentration during the stationary phase. The concentration of HAI-1 increased continuously after 9 h of cultivation (late exponential growth phase) and reached a maximal concentration of about 10 μM (10.6±2.7 μM) in the stationary phase. Thus, during the early and mid-exponential growth phases only AI-2 is present in detectable amounts, in the late exponential growth phase AI-2 predominates over HAI-1, and the stationary phase is characterized by essentially equal molar concentrations of HAI-1 and AI-2. Note that most reports on quorum sensing in *V. harveyi* have relied on cell density measurements, most commonly in the range between 10^6^ and 10^8^ cells*mL^−1^ [8], values that correspond to the early and mid-exponential growth phases (OD_600_ ranging from 0.001 to 0.1).

To estimate the productivity of the population, the measured concentrations of HAI-1 and AI-2 were normalized to the corresponding cell density (Fig. 2B). Remarkably, this revealed that the normalized AI-2 concentration actually decreases significantly when the population enters the stationary phase. In contrast, the normalized HAI-1 concentration remained constant once the maximal level was reached (Fig. 2B). These data suggest that, in the case of AI-2, *V. harveyi* either ceases to produce this AI at a certain point and/or the bacterium has other ways of reducing the number of AI-2 molecules present in the medium. The time course for HAI-1 productivity per cell corresponds to the typical threshold-mediated regulation in quorum sensing.

We were unable to determine exact concentrations for CAI-1 in cell-free culture fluids. Instead, variations in CAI-1 levels were measured using the *V. cholerae* MM920 reporter strain [33], [50]. High CAI-1 activity was detectable in the stationary phase, while only a low basal level of active CAI-1 was present during exponential growth (Fig. 3A). In parallel, *V. harveyi* JHM626 was used as reporter strain, which revealed comparable results with a basal CAI-1 activity during exponential growth and a high activity within the stationary phase (data not shown). These results were supported by GC-TOF-MS profiling (Fig. 3B and 3C). A compound which mass corresponds to that of the *V. harveyi*-specific Ea-C8-CAI was identified in the culture fluids of cells grown to the stationary phase, but not in cell-free culture fluids isolated from cultures in exponential growth phase (Fig. 3C). It is worth mentioning here that, in addition to this compound, six other metabolites (Fig. 3C) accumulated in late-stage cultures (relative to their levels in the exponential growth phase). Their chemical structures are still unknown.

In summary, the three AIs produced by wild type *V. harveyi* exhibit distinct patterns of accumulation in growing cultures. Consequently, the various growth phases are associated with different levels and blends of extracellular AI-2, HAI-1 and CAI-1.

### Induction of luminescence and exoproteolytic activity is postponed during growth of V. harveyi

The *luxCDABE* operon encoding the luciferase in *V. harveyi*, as well as a distantly located gene encoding an extracellular metalloprotease, is induced in an AI-dependent manner [18]. Bioluminescence and exoproteolytic activity were determined in samples taken from the same liquid culture of *V. harveyi* BB120 as described above. Residual bioluminescence decreases upon dilution of the cells but, after a short lag phase, bioluminescence begins to rise rapidly (Fig. 2C). This renewed onset of bioluminescence occurred at a cell density of 2.5*10^6^ cells*mL^−1^ (OD_600_ = 0.0025), which is in agreement with earlier reports [8]. It is important to note that, at this stage of growth, AI-2 is essentially the only AI present (Fig. 2A). Bioluminescence reached its maximal value in the late exponential growth phase (Fig. 2C), shortly after AI-2 peaked and HAI-1 had attained its half-maximal concentration (Fig. 2A). Thereafter, bioluminescence intensity decreased. As illustrated in Figure 2D, when normalized with respect to cell number, bioluminescence intensity displays a typical sharp decrease in the absence of AIs, an increase during the exponential growth phase, and a slow decrease thereafter.

**Figure 4:**
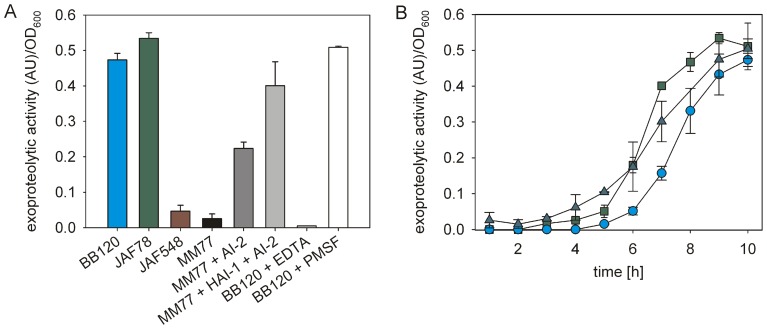
Exoproteolytic activity of *V. harveyi* mutants. (A) Exoproteolytic activity was analyzed in cell-free culture fluids of the wild type BB120 (blue) in comparison to the autoinducer-independent, constitutively active mutant JAF78 (Δ*luxO*) (green), and the quorum sensing negative mutant JAF548 (*luxO-*D47E) (red). Furthermore, the exoproteolytic activity produced by the autoinducer synthase mutant MM77 (*luxM*::Tn*5 luxS*::Cm^r^) in the absence (black) or in the presence of AI-2 (gray) or HAI-1 and AI-2 (each 10 μM) (light gray) was determined. Culture fluids were obtained from cells grown to the stationary phase. All experiments were performed in triplicate, and error bars indicate standard deviations of the mean. To classify the type of exoprotease detected, the metalloprotease inhibitor ethylenediaminetetraacetic acid (EDTA, 5 mM) (white, striped to the right) or the serine protease inhibitor phenylmethylsulfonyl fluoride (PMSF, 1 mM) (white) was added to the activity assay. (B) Time courses of the exoproteolytic activity of growing cells of strains BB120 (wild type, blue circles), JAF78 (autoinducer-independent, constitutively active mutant, green squares), and BB120 in the presence of synthetic HAI-1 (10 μM), which was added at time point 0 (dark gray triangles). All experiments were performed in triplicate, and error bars indicate standard deviations of the mean.

In the next experiment we determined the time course of the AI-dependent induction of exoproteolytic activity in cell-free culture fluids from a growing *V. harveyi* culture. Since AI-dependent induction of a gene encoding a putative exoprotease has only been described at the transcriptional level [18], it was first necessary to test whether the detectable exoproteolytic activity was indeed regulated under the control of AIs. For this purpose we analyzed the exoproteolytic activity in culture fluids of various mutants that had been grown to the stationary phase (Fig. 4A). The exoproteolytic activity measured for the wild type strain was comparable to the activity seen in the AI-independent, constitutively active mutant JAF78 (Δ*luxO*-Cm^r^). The quorum sensing negative mutant JAF548 (*luxO-*D47E), as well as the mutant MM77 (*luxM*::Tn5, *luxS*::Cm^r^) which is unable to produce HAI-1 or AI-2, exhibited very low activities. Proteolytic activity could be restored in mutant MM77 by adding both HAI-1 and AI-2 at physiological concentrations (Fig. 4A). These data confirmed that the exoproteolytic activity determined in the culture fluids of *V. harveyi* is regulated by AIs. Furthermore, this protease belongs to the metalloproteases, since it was inhibited by ethylenediaminetetraacetic acid (EDTA), but was insensitive to phenylmethylsulfonyl fluoride (PMSF) (Fig. 4A).

Analysis of a wild type *V. harveyi* population indicated that exoproteolytic activity was absent during the first 10 h of cultivation (Fig. 2C). Subsequently, activity coincided with the increase in the HAI-1 concentration, reaching a maximum in the stationary phase (after 15 h of cultivation; Fig. 2C). Normalization of the proteolytic activity to the corresponding optical density did not significantly alter the shape of the hyperbolic curve (Fig. 2D).

To test whether the appearance of HAI-1 in the medium times the induction of exoproteolytic activity, we added an excess of HAI-1 to a culture at time 0. In this case, exoproteolytic activity was first observed in the mid-exponential growth phase (at 8 h), significantly earlier than in the untreated wild type population (at 10 h) (Fig. 4B). Although HAI-1 clearly influences the onset of the induction of the exoproteolytic activity, this phenotype did not immediately develop after addition of synthetic HAI-1. Similarly, mutant JAF78 did not show constitutive exoproteolytic activity (Fig. 4B). These results unambiguously indicate the involvement of further, as yet unknown, regulatory mechanisms. These control mechanisms might be effective at the level of transcription or enzymatic activity or protein export.

In summary, induction of the exoprotease is temporally decoupled from the onset of bioluminescence, despite the fact that the corresponding genes are primarily under the control of the same signaling cascade. This notion supports the idea that different blends of AIs drive different outputs.

### Bioluminescence and exoprotease activity are the result of different AI combinations

**Figure 5:**
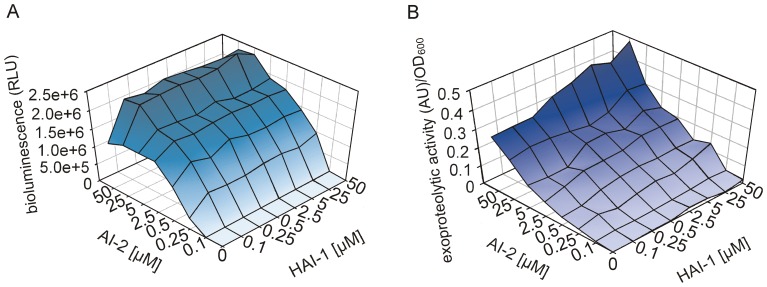
Dose-dependent effects of HAI-1 and AI-2 on bioluminescence and exoproteolytic activity of *V. harveyi.* The autoinducer synthase negative mutant *V. harveyi* MM77 (*luxM*::Tn*5 luxS*::Cm^r^) was used to analyze the dose-dependent effects of HAI-1 and AI-2. Strain MM77 was cultivated in the presence of varying concentrations (0, 0.1, 0.3, 0.5, 2.5, 5, 25 and 50 μM) of HAI-1 and/or AI-2, and levels of bioluminescence (A) and exoproteolytic activity (B) in the culture fluids were determined. Light levels and exoproteolytic activities were expressed relative to the optical density of the culture, and values are displayed in a 3D mesh. All experiments were performed in triplicate, and mean values are shown. The standard deviations were below 5%.

To experimentally test this idea we monitored the induction of bioluminescence and exoproteolytic activity in the *V. harveyi* mutant MM77 (*luxM*::Tn5, *luxS*::Cm^r^) after adding different concentrations and mixtures of AI-2 and HAI-1. Induction of bioluminescence showed a linear dependence on AI-2 concentration over the range from 0.1 to 5 μM (Fig. 5A). At very high concentrations (25 µM and 50 µM) no further increase was found.

When we tested D-HAI-1, we also found that bioluminescence was induced, albeit with much reduced efficacy. The level of bioluminescence induced by D-HAI-1 was only 0.06% of that observed following the addition of the same concentration (5 μM) of AI-2 (Fig. 5A). The functionality of the D-HAI-1 used was confirmed by *in vitro* phosphorylation experiments with LuxN and LuxU (data not shown). The L-HAI-1 isomer caused no significant induction (data not shown), which is in agreement with the known stereospecificity of *V. harveyi* HAI-1 [51]. The dark phenotype of a *luxS/cqsA* mutant, which produces only HAI-1 [8], is compatible with the low intensity of bioluminescence induced by HAI-1 observed here. By contrast, in the *luxM/luxS* mutants KM413 [26] and KM135 [18] (which are comparable to the MM77 strain used in this study), bioluminescence could be induced by HAI-1 (either by synthetic HAI-1 [26] or HAI-1 containing culture fluids [18]). It is important to note that all these mutants are able to produce CAI-1. In our experiments bioluminescence was measured of mid-exponentially grown cells, when CAI-1 was not detectable (see Fig. 3A). In former studies CAI-1 might be responsible for bioluminescence induction, because cells were analyzed after 14–16 h of growth [18], [26]. Note that, as described above, HAI-1 is at no time the sole AI to be found in a wild type culture, and our results indicate that induction of bioluminescence by HAI-1 is dependent on the presence of other AIs.

We therefore tested the effects of HAI-1 and AI-2, applied in different molar ratios, on the induction of bioluminescence (Fig. 5A). Importantly, bioluminescence increased when both HAI-1 and AI-2 were present (Fig. 5A). This effect (about 2-fold) was particularly pronounced at the lowest AI-2 concentrations tested (0.1 μM and 0.25 μM) and a low concentration of HAI-1 (0.1 μM); no further increase was observed upon exposure to higher concentrations of HAI-1. Thus, while AI-2 is able to induce bioluminescence in *V. harveyi* (1.3*10^6^ RLU, for AI-2 at 0.25 μM) on its own, the simultaneous presence of HAI-1, which has only a minor effect by itself (1.2*10^3^ RLU, for HAI-1 at 0.25 μM), significantly increases the level of bioluminescence observed (2.5*10^6^ RLU for HAI-1 and AI-2, each at 0.25 μM).

Then we tested the dose-dependent effect of AIs on the induction of the exoprotease. An increase in the AI-2 concentration led to a concomitant increase in the exoproteolytic activity. HAI-1 induced this activity too, but to a much lesser degree (between 5% and 15%; see Fig. 5B). Finally, a mixture of HAI-1 and AI-2 resulted in maximal exoproteolytic activity (Fig. 5B). These results correlate with the onset of exoproteolytic activity in a growing wild type population at a time when both HAI-1 and AI-2 are present in the medium (Fig. 2C).

### luxR transcription levels follow the pattern of AIs accumulation in a growing V. harveyi population

**Figure 6:**
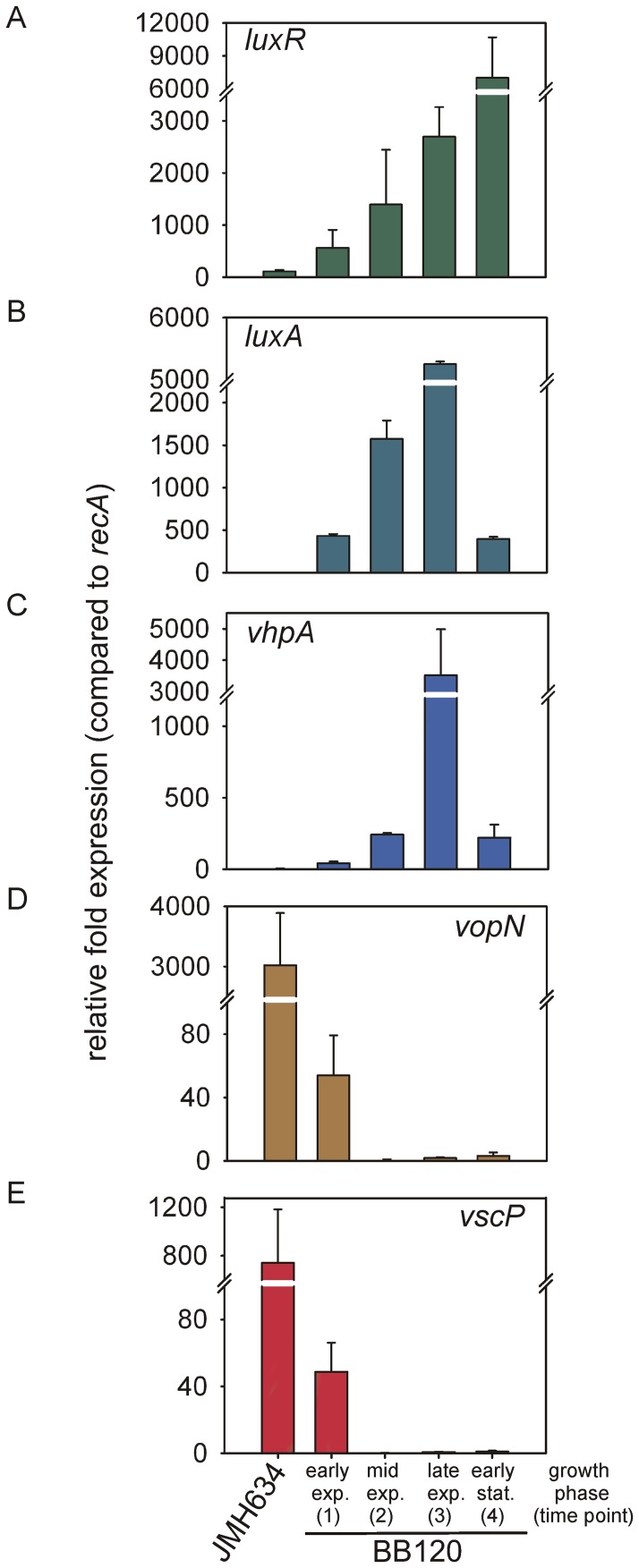
Transcriptional analysis of AI-regulated genes. Cells of the wild type (BB120) and the autoinducer-negative mutant JMH634 were grown as described in Fig. 2. Total RNA was isolated at four different time points (marked by the arrows in Fig. 2A), which are characterized by different concentrations/blends of the AIs: 1– early exponential growth phase  =  low concentration of AI-2; 2– mid-exponential growth phase  =  high concentration of AI-2; 3– late exponential growth phase  =  blend of AI-2 and HAI-1; 4– stationary phase  =  blend of AI-2, HAI-1 and CAI-1. Levels of *luxR* (A), *luxA* (B), *vhpA* (C), *vopN* (D), *vscP* (D) and *recA* (as reference) transcripts were determined by qRT-PCR for each time point. Changes in transcript levels (expressed relative to *recA*) were calculated using the C_T_ method [49]. Since transcript levels of the corresponding genes in mutant JMH634 did not change significantly over time, only one time point (3) is shown. All experiments were performed in triplicate, and error bars indicate standard deviations of the mean.

Next we analyzed the level of the transcript encoding the master regulator LuxR at different time points during growth (see arrows in Fig. 2A), which are characterized by different concentrations/blends of the AIs (1– early exponential growth phase  =  low concentration of AI-2; 2– mid-exponential growth phase  =  high concentration of AI-2; 3– late exponential growth phase  =  blend of AI-2 and HAI-1; 4– stationary phase  =  blend of AI-2, HAI-1 and CAI-1). As a control, the synthase negative mutant JMH634, which is unable to produce AI-2, HAI-1, and CAI-1, was analyzed at essentially the same stages of growth. Cells were cultivated, RNA was isolated, cDNA was synthesized, and levels of the *luxR* transcript were determined by qRT-PCR (Fig. 6A). Changes in *luxR* mRNA levels relative to the *recA* transcript were calculated using the C_T_ method [49]. The level of *luxR* mRNA in the wild type increased with the buildup in AI-2 concentration (time points 1 and 2), and rose further when HAI-1 appeared in the medium (time point 3). The maximal transcript level was measured at the time when all three AIs were present (time point 4; 54-fold induction compared to the mutant). The number of transcripts per cell (calculated according to [52]) revealed an increase from 0.9, 2.2, 4.2 to 11.0 transcripts per cell from the early exponential to the stationary growth phase. In the mutant JMH634 0.2 *luxR* transcripts per cell were detectable, indicating that *luxR* is not completely repressed in the absence of AIs. However, the effects of extremely low concentrations of LuxR on cell physiology are still unknown. The number of LuxR proteins per cell is difficult to deduce from these data, due to the numerous feedback mechanisms. Nevertheless, it is expected that the number of transcripts is reflected in the number of LuxR molecules produced (see [52] for quantitative data), which in turn is the primary parameter that determines the responses of different gene classes (Fig. 1).

### AI-regulated genes harbor different transcription profiles

Transcript levels were also determined for four AI-regulated genes [26]. The experiments were essentially the same as described above for *luxR*. The profiles for *luxA* (which codes for a subunit of luciferase), *vhpA* (an exoprotease), *vopN* (an outer membrane protein) and vscP (a putative translocation protein in type III secretion) transcripts all differed in detail (Fig. 6B–E). *luxA* was induced by up to 1,500-fold at stages when AI-2 was the major AI in the medium (time points 1 and 2; Fig. 6B). When HAI-1 became available the *luxA* transcript level increased further (time point 3; 3-fold additional increase). At time point 4 (LuxR level highest), the transcript level of *luxA* was low. Luciferase is a stable protein, which might explain the transcriptional down-regulation. Nevertheless, the drop in *luxA* transcript level coincides with the decline in bioluminescence described above (Fig. 2).

In contrast, levels of the *vhpA* transcript increased very slightly between time points 1 and 2, while the maximum value was found at time point 3, when both HAI-1 and AI-2 were present (750-fold induction at time point 3 compared to mutant JMH634; Fig. 6C). Thereafter the transcript level decreased. Increasing AI-2 concentrations are associated with increased repression of *vopN* and *vscP* (time points 1 and 2; Fig. 6D, E). HAI-1 and CAI-1 have no additional effect (time points 3 and 4, Fig. 6D, E).

In conclusion, different combinations of AIs present at certain growth stages drive different AI-regulated processes, and thus determine their timing and succession.

### HAI-1 and AI-2 act synergistically on the phosphorylation cascade of V. harveyi

**Figure 7:**
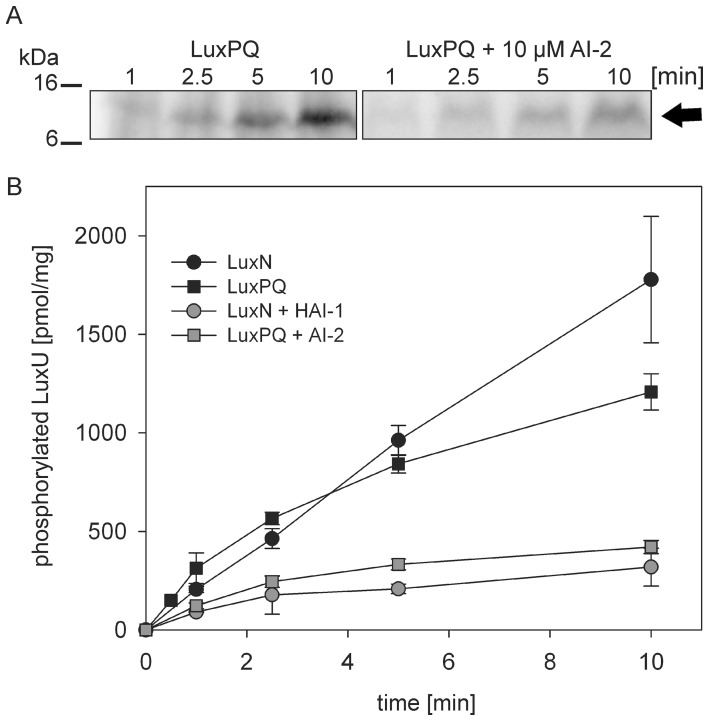
Phosphorylation activity of LuxQ. Inverted membrane vesicles prepared from *E. coli* TKR2000 containing full-length LuxQ were incubated with purified LuxP, purified LuxU and (where indicated) with 10 μM AI-2 (A). The phosphorylation reaction was started by adding 100 μM [γ-^32^P] ATP at time 0. At the indicated times, the reaction was terminated, and radiolabeled proteins were separated by SDS-PAGE, and visualized by autoradiography. The arrow indicates phosphorylated LuxU. Phosphorylated LuxU was quantified with ImageQuant using [γ-^32^P] ATP as standard (B). Phosphorylation experiments were also performed in the presence or absence of 10 μM HAI-1 using membrane vesicles containing full-length LuxN and phosphorylated LuxU was quantified accordingly (B).

We performed *in vitro* phosphorylation assays to test the effects of different inputs, specifically, different ratios of HAI-1 and AI-2, on the LuxN and LuxQ (LuxP)-mediated phosphorylation of LuxU as output. The full-length hybrid kinases LuxN and LuxQ (tagged with 6 histidine residues) were expressed in the *E. coli* strain TKR2000. This strain lacks the F_1_/F_o_-ATPase, and inverted membrane vesicles can be used directly for phosphorylation experiments. Analogously to a biochemical characterization of the HAI-1-recognizing kinase LuxN described earlier [14], an initial characterization of the AI-2-sensing LuxQ in interplay with LuxP (LuxPQ) was performed (Fig. 7). Western blot analysis using purified protein revealed that LuxN and LuxQ were incorporated into the lipid bilayers of membrane vesicles, and accounted for about 2.7% and 1.8% of all membrane proteins (data not shown). Since the LuxQ-LuxP interaction does not change in the presence of AI-2 [10], all studies were performed with LuxQ and purified LuxP in a molar ratio of 1∶1. LuxPQ was able to phosphorylate LuxU in a time-dependent manner (Fig. 7A). The LuxPQ kinase activity was determined to be in the same range as the LuxN kinase activity (initial rates 300 and 200 pmol*min^−1^*mg^−1^ kinase protein, respectively) (Fig. 7B). Addition of AI-2 inhibited the LuxPQ kinase activity (Fig. 7) in a concentration-dependent manner (data not shown), with half-maximal inhibition occurring at 5 μM AI-2. Importantly, even at the highest AI-2 concentration tested (30 μM), LuxU phosphorylation was still detectable (data not shown). These findings are reminiscent of the incomplete inhibitory effect of HAI-1 on the LuxN kinase activity [14]. When each AI was added to its cognate kinase at a concentration of 10 μM, LuxU phosphorylation by LuxPQ and LuxN was inhibited to comparable extents (Fig. 7B, 61% and 57%, respectively). Moreover, HAI-1 had no effect on LuxPQ-mediated phosphorylation of LuxU, and AI-2 had no effect on LuxN-mediated phosphorylation of LuxU (data not shown). LuxPQ also catalyzed the dephosphorylation of phospho-LuxU, and this reaction was unaffected by the presence of AI-2 (data not shown).

**Figure 8:**
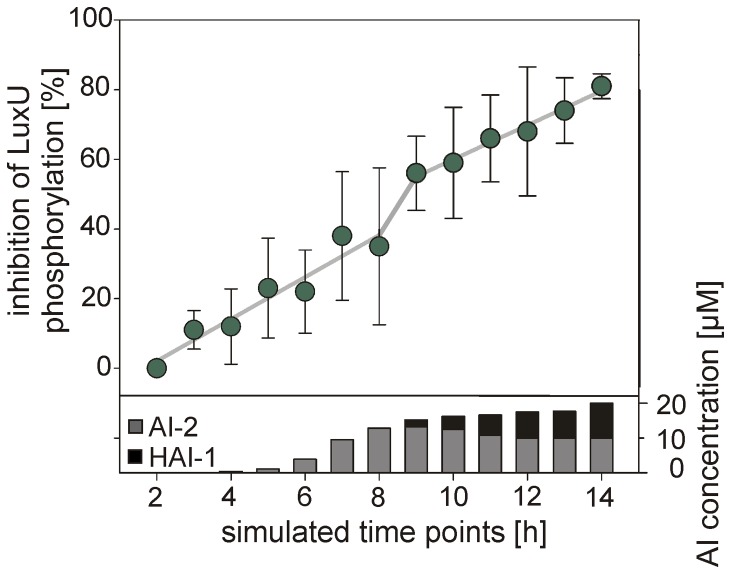
Effects of various concentrations of HAI-1 and AI-2 on LuxN- and LuxQ- mediated phosphorylation of LuxU. LuxN- and LuxQ-bearing membrane vesicles, together with purified LuxP and LuxU, were incubated with 100 μM [γ-^32^P] ATP, and the effects of AI-2 and HAI-1 on the initial rate of LuxU phosphorylation were tested. AI-2 and HAI-1 were added at physiological concentrations (see Fig. 2), indicated in the lower part of the graph (HAI-1 in black, AI-2 in gray). Phosphorylated LuxU was quantitatively analyzed as described in Fig. 7. The degree of inhibition is expressed as the percentage reduction in the initial rate of LuxU phosphorylation measured in the presence of the indicated concentrations/blends of autoinducers relative to that seen in the absence of autoinducers. All experiments were performed in triplicate, and error bars indicate standard deviations of the mean.

In order to simulate the situation *in vivo*, we designed an experiment in which the total rate of LuxN and LuxPQ (molar ratio LuxN:LuxQ  = 1∶1) mediated LuxU phosphorylation was assayed in the presence of various combinations of AIs. In so doing, we utilized the physiological concentrations of HAI-1 and AI-2 we had found to be present in a growing wild type culture *in vivo* (Fig. 2). In the absence of AIs, LuxU was readily phosphorylated (initial rate of 250 pmol*min^−1^*mg^−1^ kinase protein, 100%). Addition of increasing amounts of AI-2 led to concomitant inhibition of LuxU phosphorylation (Fig. 8, simulated time points 1–8 h). Upon supplementation with HAI-1, a significant increase in inhibition (from 35% to 50%) was observed (Fig. 8, simulated time points 8 and 9 h). Moreover, the use of HAI-1 and AI-2 in ratios characteristic of longer cultivation times resulted in a linear increase in inhibition although the slope was lower than for AI-2 alone (Fig. 8, simulated time points 9 to 14 h). However, the highest combined concentration of the two AIs (HAI-1:AI-2 = 1∶1) tested was insufficient to completely inhibit LuxN/LuxPQ-mediated phosphorylation of LuxU (Fig. 8). These findings thus leave room for the input of the third (the CAI-1 responsive CqsS) and fourth (the NO-sensing H-NOX/HqsK) systems. Unfortunately, synthetic CAI-1 is not commercially available and therefore could not be included in the phosphorylation experiments thus far.

In conclusion, the sensory part of the complex signaling cascade responds sensitively to various concentrations and blends of AIs by generating distinct outputs at the level of phosphorylated LuxU. Subsequently, these signals are transduced by the same signaling cascade via LuxO and Qrr to *luxR*, which permits fine-tuning of the level of the *luxR* transcript (Fig. 6A) and thus enables tight control of LuxR-regulated genes.

## Discussion

Like *V. harveyi*, other bacterial species also use more than one AI for quorum sensing. For example, *Staphylococcus aureus* [53], [54] and *Vibrio cholerae* [55] produce and respond to two, *Pseudomonas aeruginosa* [56] and *Aliivibrio fischeri* [55], [57] to three different AIs. Here we report time and growth phase-dependent alterations in the onset and concentration of each of the three *V. harveyi* AIs in liquid culture. Importantly, during the shift from low to high cell density (10^6^ to 10^8^ cells/mL, OD_600_ = 0.001 to 0.1) that occurs in the exponential growth phase, AI-2 is the major AI in the medium. HAI-1 first becomes detectable and increases in concentration during the late exponential growth phase. Under our growth conditions, CAI-1 was detected at readily measurable levels only in the stationary phase. Since we were unable to determine the molar concentration of CAI-1, the sensitivity of our method is unknown. Therefore, we cannot exclude that physiological relevant low concentrations of CAI-1 are present at earlier growth phases. The delay in secretion of HAI-1 relative to AI-2 is in good agreement with data in a recent report [57], although the two sets of results are not strictly comparable, because different growth media were used (the more complex LM medium in [57], AB medium here). Moreover, a previous study has shown that CAI-1 activity peaks in the stationary phase when cells are grown in LM medium [8], in agreement with our observations. These data show that different stages in the expansion of a *V. harveyi* culture are characterized by distinct AI profiles: the early exponential growth phase by low AI-2, the mid-exponential growth phase by high AI-2, the late exponential growth phase and the early stationary phase by a blend of AI-2 and HAI-1, and the later stationary phase by a combination of AI-2, HAI-1 and CAI-1. This classification corresponds well with the staggered expression of bioluminescence and exoproteolytic activity during growth of wild type *V. harveyi*. Although both phenotypes are dependent on AI-controlled genes and hence on the same signaling cascade, they are not induced simultaneously. The onset of bioluminescence occurs, and light levels reach their maximum, in the exponential growth phase, whereas exoproteolytic activity only sets in after the transition into the stationary phase. These findings are supported by our reporter strain analysis, which indicated that AI-2 is sufficient for induction of bioluminescence and that HAI-1 (even at a low concentration) acts synergistically to enhance light production. In contrast, both HAI-1 and AI-2 were required to induce exoproteolytic activity. Other AI-regulated phenotypes seem also to be affected by different combinations of AIs. Based on our experiments, full repression of *vscP* and *vopN* requires only AI-2. Furthermore, the sRNA Qrr4 can be induced by HAI-1 or AI-2, but full induction is attained only when both are present together [39]. The effects of HAI-1 and AI-2 on the promoter activities of AI-regulated genes have been analyzed previously using promoter::*gfp* fusions [26], and these studies permitted differentiation between three groups of genes. The first group requires both AIs for activity; either HAI-1 or AI-2 can induce the second set, but both are necessary for full activity, and either HAI-1 or AI-2 is sufficient to induce full activity of the third. Remarkably, we observed a tight correlation between the various inputs and the level of the *luxR* transcript that encodes the master regulator of the signaling cascade. With each additional AI, levels of *luxR* mRNA increased. The highest level was measured when all three AIs were present simultaneously. Curiously, no gene is yet known to be regulated by LuxR at this late growth stage.

LuxR activates and represses more than 100 genes, and both the numbers and relative affinities of its binding sites vary for different genes [15]. The level of extracellular AIs as input is translated into a particular intracellular concentration of LuxR. A low LuxR concentration in the cell seems to be sufficient for the induction of *luxA* and hence for bioluminescence, and for the repression of *vopN* or *vscP*. At later growth stages, levels of the *luxR* transcript increase, and *vhpA*, which codes for a protease, is induced to a maximal level. In agreement with this, full induction of the exoproteolytic activity requires both HAI-1 and AI-2, and hence a higher copy number of LuxR than does the induction of bioluminescence.

The transcriptional analysis raises questions regarding the molecular mechanism of down-regulation of gene expression. For example, significantly decreased transcript levels were determined for *luxA* and *vhpA* during stationary phase. It is still unclear whether LuxR or AphA [24] – a transcriptional regulator that acts in the opposite manner to LuxR – or other components of the stationary phase control network are responsible for this phenomenon.

Our *in vitro* data on receptor-mediated phosphorylation of LuxU, the protein which gathers all information, reveal a very tight correlation between various inputs and outputs. The rate of LuxU phosphorylation decreases linearly with the physiological increase in the AI-2 concentration, and the decrease continues as HAI-1 is added to the mix. Remarkably, the activities of the two histidine kinases LuxN and LuxPQ exhibit some degree of cooperativity, because the effects of AI-2 and HAI-1 were non-additive (Fig. 8). Even at a low concentration, HAI-1 had a significant effect on the inhibition of LuxU phosphorylation (Fig. 8). Furthermore, the blend of AI-2 and HAI-1 available in the late stationary phase did not suffice to prevent LuxU phosphorylation, indicating that the system has capacity to spare for the integration of information, e.g. from the CAI-1/CqsS and NO H-NO/HqsK circuits. Cooperativity between the different histidine kinases is supported by earlier *in vivo* measurements with mutants lacking one or two histidine kinases [8]. Mutants lacking either LuxN or CqsS or the corresponding double mutant required a higher cell density (according to our data a higher AI-2 concentration) to induce bioluminescence. In contrast, in a mutant lacking LuxQ, a lower HAI-1 and/or CAI-1 concentration was sufficient for luminescence induction. Thus, deletion of kinases has a greater or lesser effect on the sensitivity of the quorum sensing system depending on the AIs to which each responds.

The *in vitro* data also complement a comprehensive study on input-output relationships in various feedback-loop mutants [25]. There, it was clearly demonstrated that feedbacks affecting the cellular concentrations of LuxR as well as LuxO ensure a broad and graded response to HAI-1 and AI-2, and prevent switch-like on-off behavior. Here we found that the receptor-mediated input ensures a graded output already at the level of phosphorylated LuxU. Thus far, our *in vitro* studies have used equal quantities of LuxN and LuxPQ. In future experiments we will integrate the other histidine kinases, and test different ratios of the histidine kinases to take into account the recently described positive *luxMN* feedback loop and the increased sensitivity to HAI-1 [25].

The stable succession of different AI-regulated processes might facilitate the proliferation of *V. harveyi* in the ocean. Bioluminescence might attract organisms of the same species to form aggregates or to settle down on surfaces. *V. cholerae* is known to possess blue-light photoreceptors [58]. Based on genome analyses, *V. harveyi* also possesses genes encoding proteins with a BLUF domain, a sensor for blue light. Bioluminescence improves the nutrient cycle [59] as well as the metabolization of oxygen, and thereby reduces the number of oxygen radicals [60], [61]. In this way microcolonies could benefit from light production during the infection of shrimps. In addition, *V. harveyi* might use additional AI-2 that is produced by other species. Later, when its population has reached a certain cell density, *V. harveyi* produces and responds to the species-specific HAI-1. Subsequently, HAI-1 boosts bioluminescence induction. At this growth stage, which coincides with stationary growth and the beginning of biofilm formation [17], the population starts to produce an exoprotease. Exoenzymes might be useful for the recycling of dead cells during stationary growth or for the release of single cells from aggregates. Exoproteases are also important for the pathogenicity of some *Vibrio* species [62], [63]. By utilizing the species-specific HAI-1 to induce the exoprotease, *V. harveyi* ensures that the products of exoproteolysis are made available to its own kind. Unfortunately, no gene is known which is under the control of CAI-1 in the stationary phase. Nonetheless, it is suggested that *V. harveyi* needs all three AIs to time the onset and duration of certain AI-regulated processes during different stages of growth.
